# Supplementary material for: Expression of Talaromyces marneffei acuM and acuK Genes in Gluconeogenic Substrates and Various Iron Concentrations
Source: J Fungi (Basel). 2020 Jul 8;6(3):102. doi: 10.3390/jof6030102 (PMC7558521; doi:10.3390/jof6030102)
Supplement: Supplementary file 1 [file jof-06-00102-s001.pdf]

Supplement data

Method

Bioinformatics for characterization of *acuM* and *acuK* genes in *Talaromyces marneffei*

DNA sequences of *acuM* and *acuK* genes were obtained from a BLAST search with the whole genome sequencing project of *Talaromyces marneffei* ATCC18224 (Genbank database). The search was performed by using accession number for *acuM* and *acuK* from *Aspergillus fumigatus*, Afu2g12330 and Afu2g05830 as the query sequences. Then part of the contigs containing *acuM* and *acuK* genes of *T. marneffei* were analyzed to identify the open reading frame, transcript, and deduce the amino acid sequences by using FGENESH program (http://www.softberry.com). The homology sequence analysis of AcuK and AcuM deduced amino acids were perfume by BlastP web-based analysis program (www.ncbi.nlm.nih.gov/blastp). The box alignment and phylogenetic tree were extracted from the BlastP analysis.

Result

Characterization of the *acuM* and *acuK* genes in *Talaromyces marneffei*

The genes encoding AcuK and AcuM in *T. marneffei* were identified from the whole genome sequence database of *T.marneffei* (ATCC18224) and analyzed. The *acuM* gene encompassed seven exons. The transcript length was 1,617 bases and encoded a polypeptide of 538 amino acids. The *acuK* gene contains three exons encoding a polypeptide of 674 deduced amino acids (Fig. 1A). The AcuK and AcuM proteins in *T. marneffei* are Zn(2)Cys(6) transcription factors. The conserved sequences for the DNA binding motif are shown in Fig. 1B. BlastP analysis of the deduced amino acids from *acuK* and *acuM* showed that the homologous proteins were found only in filamentous ascomycetes fungi in

genus *Talaromyces, Aspergillus*, *Penicillium*, *Trichophyton* and *Coccidoides* (Fig. 1C and D). However, most of them have not been functional characterization except for *A. nidulans* and *A. fumigatus*.

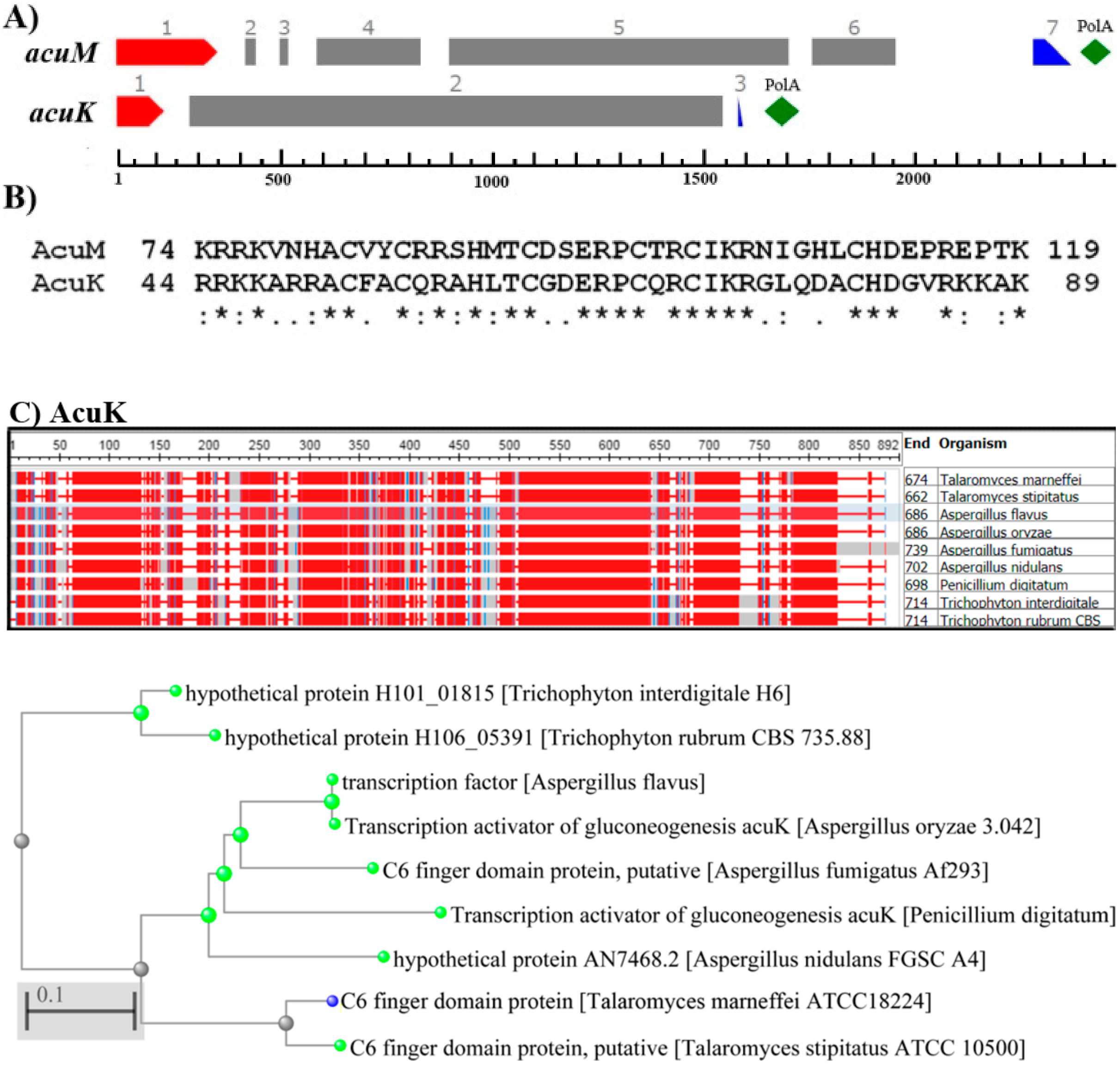

D) AcuM

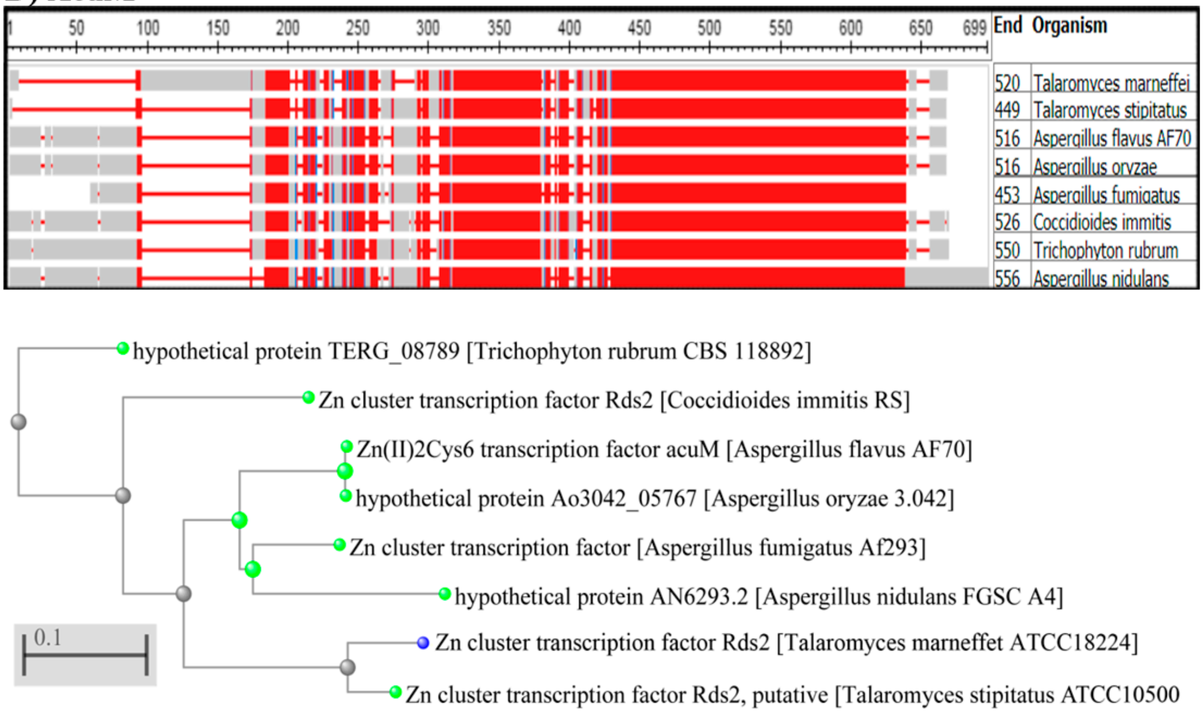

Figure S1 - Analysis of the *acuM* and *acuK* genes in *Talaromyces marneffei*. (**A**) The organization of genes showing exons and polyadenylation site (PolA) (**B**) The Zn(2)Cys(6) binuclear cluster DNA-binding domain in the AcuK and AcuM deduced amino acid sequences. Identical residues are indicated by asterisks and similar amino acids by full-stops and colons. The sequences were aligned using an online tool clustal omega (https://www.ebi.ac.uk) (**C**) and (**D**) Homology of AcuK and AcuK among Ascomycetes fungi were shown by BlastP analysis (www.ncbi.nlm.nih/blastp). The selected homolog proteins were aligned in box representation and shown in phylogenetic tree. These diagrams were generated and included from the result of BlastP analysis.
